# Regulation of Magnesium Matrix Composites Materials on Bone Immune Microenvironment and Osteogenic Mechanism

**DOI:** 10.3389/fbioe.2022.842706

**Published:** 2022-03-14

**Authors:** Xiaojing Nie, Xueyan Zhang, Baozhen Lei, Yonghua Shi, Jingxin Yang

**Affiliations:** ^1^ Department of Pathology, School of Basic Medical Sciences, Xinjiang Medical University, Urumqi, China; ^2^ Beijing Engineering Research Center of Smart Mechanical Innovation Design Service, Beijing Union University, Beijing, China; ^3^ College of Robotics, Beijing Union University, Beijing, China

**Keywords:** magnesium, functionalmaterials, coating, osteogenesis, osteogenic mechanism, bone immunity

## Abstract

Despite magnesium based metal materials are widely used in bone defect repair, there are still various deficiencies, and their properties need to be optimized. Composites synthesized with magnesium based metal as matrix are the research hotspot, and the host immune response after biomaterial implantation is very important for bone binding. By studying the immunoregulation of bone biomaterials, it can regulate the immune response in the process of osteogenesis and create a good local immune microenvironment, which is conducive to biomaterials to reduce inflammatory response and promote good bone binding. This article introduces the osteogenic mechanism of magnesium based metal materials and its regulation on bone immune microenvironment in detail.

## Introduction

### Applications of Magnesium(Mg) Matrix Composites

Mg is a light metal with low density, high strength/weight ratio, in addition Mg and its alloys exhibit good biocompatibility as a kind of degradable and absorbable biomaterials (Qi et al., 2019). Compared with metallic materials that are now widely used in the clinic, the elastic modulus of Mg based metallic materials is closer to the bone cortex, structurally and mechanically similar to trabecular bone, favoring the growth of bone tissue toward its interior and obtaining early fixation effects, thus reducing the “stress shielding effect”. However, Mg based metal is a double-edged sword as a biomaterial. Due to the poor corrosion resistance, Mg alloys degrade too fast and do not match the time of bone tissue healing, making it difficult to exert the osteogenic effect of Mg alloys, and the gas generated during the degradation process may cause a local inflammatory response, which greatly limits the application of Mg alloys in the clinic. There are many methods to reduce the degradation rate of Mg metal materials, such as Mg purity, alloying components, surface modification and increasing coatings (Kato et al., 2019).

#### High Purity Mg

Since the purity of Mg metal reached higher (99.99% or 99.999%), it can have a degradation rate suitable for the internal fixation material of bone repair. At the same time, high-pure Mg *in vivo* degradation will not release other alloying elements, avoiding the disadvantages of other non degradable elements aggregation *in vivo*, these advantages make the application of high-pure Mg in bone repair gradually attract attention. In the related research process, it was found that pure Mg has a series of problems, such as pitting corrosion, degradation rate faster so that hydrogen gas accumulation around the implant delays the healing of the tissue (Mahapatro et al., 2015; Siddiqui et al., 2019).

#### Mg Alloys

Alloying is a general and effective method to improve the corrosion resistance and mechanical properties of Mg, and considerable work has focused on this method, such as the addition of aluminum (Al), zinc (Zn), strontium (Sr), yttrium (Y), and cerium (Ce) elements (Alvarez-Lopez et al., 2010; Seitz et al., 2015; Wu et al., 2016). However, the use of some alloying elements is still potentially hazardous to human body, such as the high concentration of Ce, the hepatotoxicity of Y as well as the neurotoxicity of Al (Zain et al., 2012). As a neurotoxicant, the accumulation of Al is associated with various neurological diseases, such as dementia, senile dementia and Alzheimer’s disease (Wang et al., 2017a).

Calcium (Ca) is a major component of bone, and Ca ions are also necessary for each cell they regulate various biochemical reactions *in vivo*, such as in the absence of serum in culture medium, and the addition of Ca ions can enhance the proliferation of stem cells and induce osteogenic differentiation (MarmionCeline et al., 2014; Lei et al., 2015). Multiple *in vitro* studies have also reported the enhancing effects of Mg or Ca on osteoblast activity, such as improving cell growth status and alkaline phosphatase (ALP) expression (Streckova et al., 2018).

Both oral and *in vitro* studies confirm that Sr can pharmacologically increase bone formation and decrease bone resorption at the cellular level (Reginster et al., 2010; Reginster et al., 2013) and Sr compounds can activate osteoblast and osteoclast activity through different signaling pathways (Saidak and Marie, 2012). Li studies proved that Sr can promote osteogenic differentiation of stem cells, and up regulation of several osteogenic genes as well as an increase in ALP protein expression was observed when rat mesenchymal stem cell (MSCs) were cultured in Sr supplemented medium (Li et al., 2012). An increase in the gene and protein expression of various osteogenic markers was detected in osteogenic human stem cells (hMSCs) cultures treated with Sr by Sila et al. (Munisso et al., 2012). In addition to this small amount of strontium can promote the regeneration of grains and thus improve the mechanical properties of pure Mg (Brar et al., 2012).

#### Surface Modification/Coating Treatment of Mg Alloy

Pure Mg or Mg alloys can be treated physically or chemically to form a “protective film” on their surface that can reduce the degradation rate of the substrate, which can include micro arc oxidation coating (MAO), chemical conversion coating, electroplating coating, biomimetic deposition coating, polymer coating, etc. (Lin et al., 2013). Among all these methods, MAO is a promising new surface treatment method, and the MAO technique shows excellent adhesion to the base metal. In addition, this technique has many advantages, such as simple surface pretreatment, simple process, low cost, and comprehensive properties, which can improve corrosion resistance, wear resistanceand microhardness of Mg alloys (Zhu et al., 2016). In immersion tests, the mechanical properties of MAO coated samples remain unchanged and have good biocompatibility. However, MAO coatings on Mg alloys are composed of micro pores and cracks, which may affect the protective properties of the coatings and accelerate the corrosion rate (Dima et al., 2014; Golshirazi et al., 2017). The coating process of the self-assembly technique resulted in the formation of a double-layer PEI/KC: Mao coating on the surface of AZ91 Mg alloy, which led to an increase in the thickness of the coating and improved the coating and filling of pores, and the bonding strength of the double-layer PEI/KC: MAO coating was significantly higher (about 1.3-fold) than that of the MAO coating, the double-layer PEI/KC: MAO coating improved the anti-corrosion ability of AZ91 Mg alloy with the potential for application in biomedicine by prohibiting the corrosive ion transport. In some studies, zirconia (ZrO_2_) materials have been used in biomedicine to obtain implants or various coatings. It is bioneutral, and its result is equivalent to titanium, but it does not affect the growth rate of osteoblasts. At the same time, ZrO_2_ nanoparticles have antibacterial properties. ZrO_2_ is an important biomaterial, widely used in applications such as dental implants, where osteointegration is of minor importance compared to the requirements. The effects of 100 nm thick titanium dioxide (TiO_2_), ZrO_2_ and hafnium oxide (HfO_2_) coatings on the corrosion behavior and cytotoxicity of AZ31Mg alloy were evaluated. The results show that ZrO_2_ has the higher corrosion resistance and cell viability (Peron et al., 2021).

#### Other Types of Composites

Recent studies have shown that customized poly lactic-co- glycolic acid (PLGA)/MgO alendronate microspheres are used to study the bone immune regulation of extracellular bioactive cations (Mg^2+^) in the bone tissue microenvironment. The microspheres give a controllable release of Mg^2+^. The results show that Mg^2+^ controlled tissue microenvironment can enhance anti-inflammatory (IL-10) and promote bone protein (BMP-2 and TGF-β1). The production of cytokines effectively induced macrophages to polarize from M0 phenotype to M2. It can also produce a good bone immune microenvironment. The newly formed bone tissues in the Mg TME possess a superior microstructure, bone mineral density, and mechanical property (Lin et al., 2021). In addition, injectable *in-situ* formed hydrogels have been extensively studied in bone regeneration applications. This hydrogel can not only be quickly and locally formed in any geometric target, but also play an important role in minimally invasive treatment, and it is also used to enhance the damage of bone tissue as a carrier for delivery of therapeutic drugs and cells. Gelatin, collagen, gelatin, hyaluronic acid and alginate, injectable hydrogels, which are prepared from natural polymers, have been widely used in bone tissue engineering due to their physical and chemical properties, excellent biocompatibility and structural similarity with extracellular matrix. Studies have shown that Injectable *in-situ* formed hydrogels as a carrier of Mg matrix composites helps to promote osteoblast differentiation (Chen et al., 2021).

## The Role of Mg Matrix Composites in Macrophage-Mediated Inflammatory Response

**FIGURE 1:**
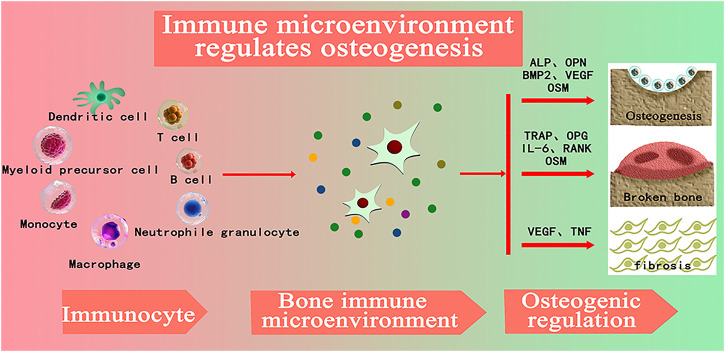
(ALP: alkaline phosphatase; OPN: Bone bridge egg white, BMP-2: Bone morphogenetic protein-2, VEGF: vascular endothelial growth factor, OSM: tumor suppressant M, TRAP: Tartrate-resistant acid phosphatase, OPG: osteoprotectin; Il-6: Interleukin-6, RANK: nuclear factor κβ receptor activation factor, TNF: Tumor necrosis factor) (Chen et al., 2018)_._

Biomaterials trigger a foreign body response (FBR) upon implantation, involving a series of inflammatory events and repair processes including protein adsorption, immune cell infiltration, cytokine and chemokine release, cell recruitment, and fiber encapsulation (Zhang et al., 2016). Therefore, the study of “bone immune microenvironment” of bone repair materials is a major issue to be faced. Timely and appropriate protection and adequate immune response will promote the integration of implant surface and host bone tissue, creating a microenvironment that promotes osteogenesis, which is conducive to promoting bone repair of implant materials (Ping et al., 2021). The development of bone immunology has become one of the greatest achievements in the field of bone biology, which reveals the important role of immune cells in regulating bone dynamics (Cui et al., 2018). By studying the immune regulation of bone biomaterials, it can regulate the immune response in the process of osteogenesis, create a good local immune microenvironment, and help biomaterials reduce inflammatory response and promote good bone binding (Haggerty et al., 2018). As shown in Figure 1, with the introduction of the concept of bone immunity and its development in the field of bone regeneration, researchers gradually found that immune cells play a central role in the formation of local bone microenvironment. By regulating the expression of growth factors, chemokines, inflammatory factors and other factors, osteogenic differentiation, osteoclast differentiation, fibrosis, vascularization and other processes closely related to bone regeneration are regulated in bone regeneration. Moreover, immune cells are the central regulators of bone immune microenvironment, and affect the process of osteogenesis, osteofragmentation and fibrosis in the process of bone regeneration by secreting various cytokines into the bone regeneration microenvironment (Chen et al., 2018).

### Polarization of Macrophage Phenotype Plays a Central Role in the Regulation of Inflammatory Response

**FIGURE 2:**
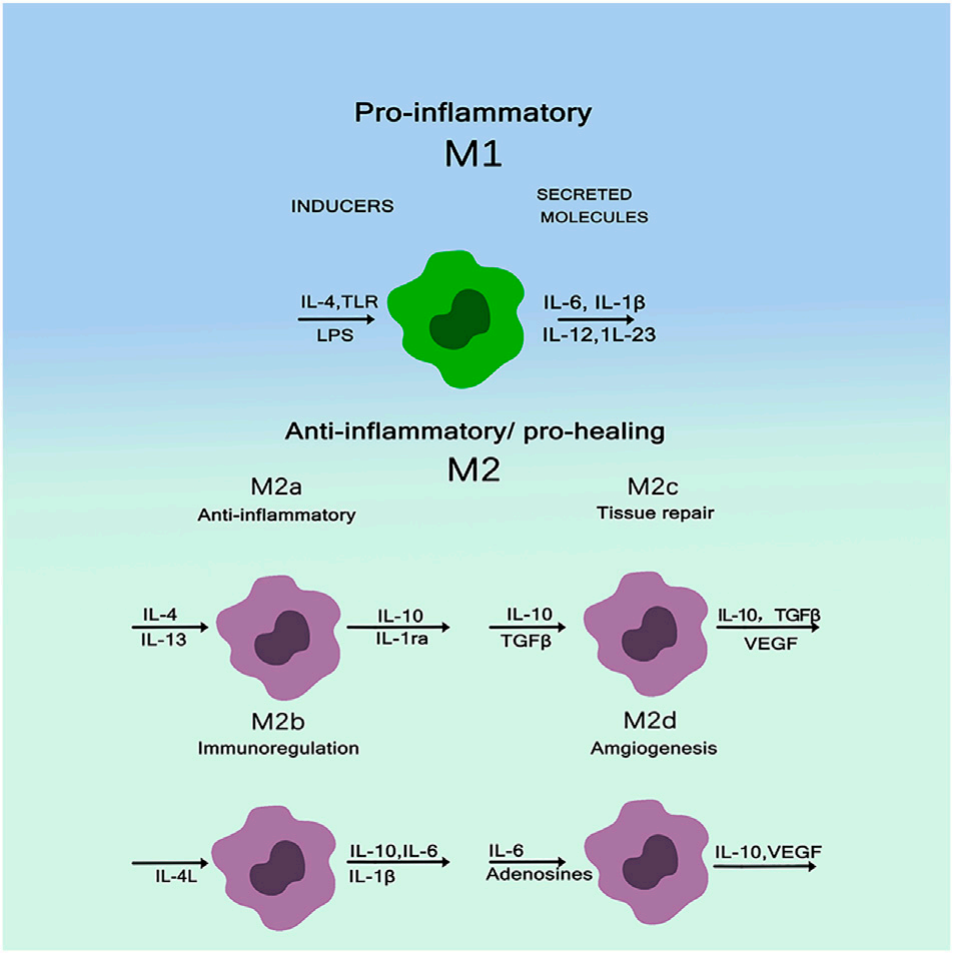
Macrophages have high plasticity and exhibit pro-inflammatory M1 or anti-inflammatory M2 types (Italiani and Boraschi, 2014)_._

Macrophages, especially monocyte derived macrophages, are key regulators. Traditionally, these cells have been known to remove foreign bodies and cell debris—both pathogens and tiny matter. Increasingly, they are found to play important roles in a wide range of processes from host defense, inflammation, wound healing, and tissue remodeling (Nenasheva et al., 2020). When exposed to external stimuli, Macrophages are activated into different phenotypes and secrete various chemokines, inflammatory mediators, matrix metalloproteinases (MMPs), and growth factors (Zhao et al., 2020; Sharma et al., 2020). Among all cells in the immune system, macrophages are innate immune cells and play an important role in the immune response induced by substances. Macrophages participate in bone physiological processes and secrete some important regulatory molecules to influence bone remodeling (Jetten et al., 2013). Biomaterial implants induced polarization of macrophages towards different phenotypes M1 and M2. M1 is a pro-inflammatory macrophage that can be “classically activated” and M2 is an anti-inflammatory macrophage that can be “selectively activated” to its microenvironment (Murray et al., 2014; Piccolo et al., 2017). Figure 2 shows macrophages are usually (induced by TH1 cytokines such as IFN-G and LPS) or selectively activated (induced by TH2 cytokines such as IL-4/IL-13), called M1 or M2, respectively. M1 macrophages polarize to perform their pro-inflammatory and pathogen-killing functions, secreting high levels of TNF-α, IL-12, and IL-23. M2 macrophages resolve inflammation and organize tissue repair, producing IL-10 and arginase. M2 are further subdivided into M2a (IL-4 and IL-13 triggers), M2b (FcgR/TLR triggers), and M2c (IL-10, TGF-β, glucocorticoid triggers). All of these subtypes have anti-inflammatory properties. While M2a and M2b are more immunomodulatory, M2c cells are considered as inactivated macrophages and participate in tissue remodeling (Italiani and Boraschi, 2014; Nahrendorf and Swirski, 2016). The interaction between macrophage polarization and biomaterial composition significantly affects bone regeneration. Thus, macrophages can be used as a cellular model to evaluate the osteogenic process of biomaterials implanted *in vitro*. Unlike osteomas, inflammatory macrophages are established during fetal development and are derived from monocyte precursors circulating in the blood. Macrophages have high plasticity and exhibit pro-inflammatory M1 or anti-inflammatory M2 type depending on the polarization phenomenon according to microenvironmental cues. M1 cells are involved in acute inflammation and, therefore, they are more closely related to the early stages of tissue repair. In contrast, M2 cells play a role in the later stages of bone healing, leading to positive repair or fibrous tissue production. Over time, the two types of macrophages release different factors and cytokines that interact with other coordinating factors of bone healing, such as endothelial cells, mesenchymal cells, osteoclasts and osteoblasts (Moeser et al., 2011; Wu et al., 2013). It is noteworthy that both M1 and M2 are required to restore the function of injured bone tissue, and timely switching between these two phenotypes may determine the success of active bone regeneration and implant.

### Regulatory Role of Mg in Macrophage-Mediated Inflammatory Response

Studies have shown that Mg^2+^, a major degradation product of Mg, is the most abundant divalent cation in cells, which can regulate cell growth, metabolism, proliferation and other cellular functions, and has the function of anti-inflammatory regulation of bone immune microenvironment (Bockstaller et al., 2005; Lee et al., 2016). For example, Mg^2+^ is used to improve the prevention of obstetric epileptic seizures. Mg^2+^ promote the conversion of macrophages to the M2 phenotype, and M2 macrophages also produce growth factors, including transforming growth factor (TGF-β) and VEGF, which support the migration and osteogenic differentiation of MSC. In addition, the inflammatory NF-κβ signaling pathway was down-regulated by inhibiting TLR pathway, and the activated macrophages were promoted to secrete anti-inflammatory cytokines IL-10 and IL-1, and the expressions of osteogenic genes BMP2 and VEGF were up-regulated. Suitable concentration of Mg^2+^ (100 mg/L) can promote osteogenic differentiation of MSC. The mechanism may be that Mg^2+^ promote the secretion of BMP2 by macrophages, by activating the BMP2/Smad signaling pathway of MSC to enhance its osteogenic differentiation ability. Mg^2+^ release concentration (about 100 mg/L) can overcome the harmful bone immunomodulatory properties of Mg-based biomaterials and make them more favorable for bone marrow. Specifically, microscale Mg^2+^induces phenotypic switching in M2 macrophages, and most likely induces an anti-inflammatory environment by inhibiting the TLR-NF-κβsignaling pathway. Microscale Mg^2+^ stimulates macrophage BMP-2 expression and activates BMP-2 signaling pathway in bone MSC, promoting osteogenic differentiation. Therefore, manipulating the concentration of in Mg-based bone scaffolds can make biomaterials have good bone immunomodulatory properties. In short, Mg matrix composites promote the transformation of macrophages to M2 phenotype, and M2 macrophages can produce TGF-β and VEGF, supporting the migration and osteogenic differentiation of BMSC and down regulate inflammatory NF by inhibiting TLR pathway-κB signaling pathway promotes activated macrophages to secrete anti-inflammatory factors IL-10 and IL-1ra, and up regulates the expression of osteogenesis related genes BMP2 and VEGF to promote osteogenesis (Mele et al., 2014).

## Mechanisms of Osteogenesis of Mg Matrix Composites

### Promotion of ERK Signaling Pathway in Osteoblasts by Degradation Products From Mg-Ca-Sr Alloys

**FIGURE 3:**
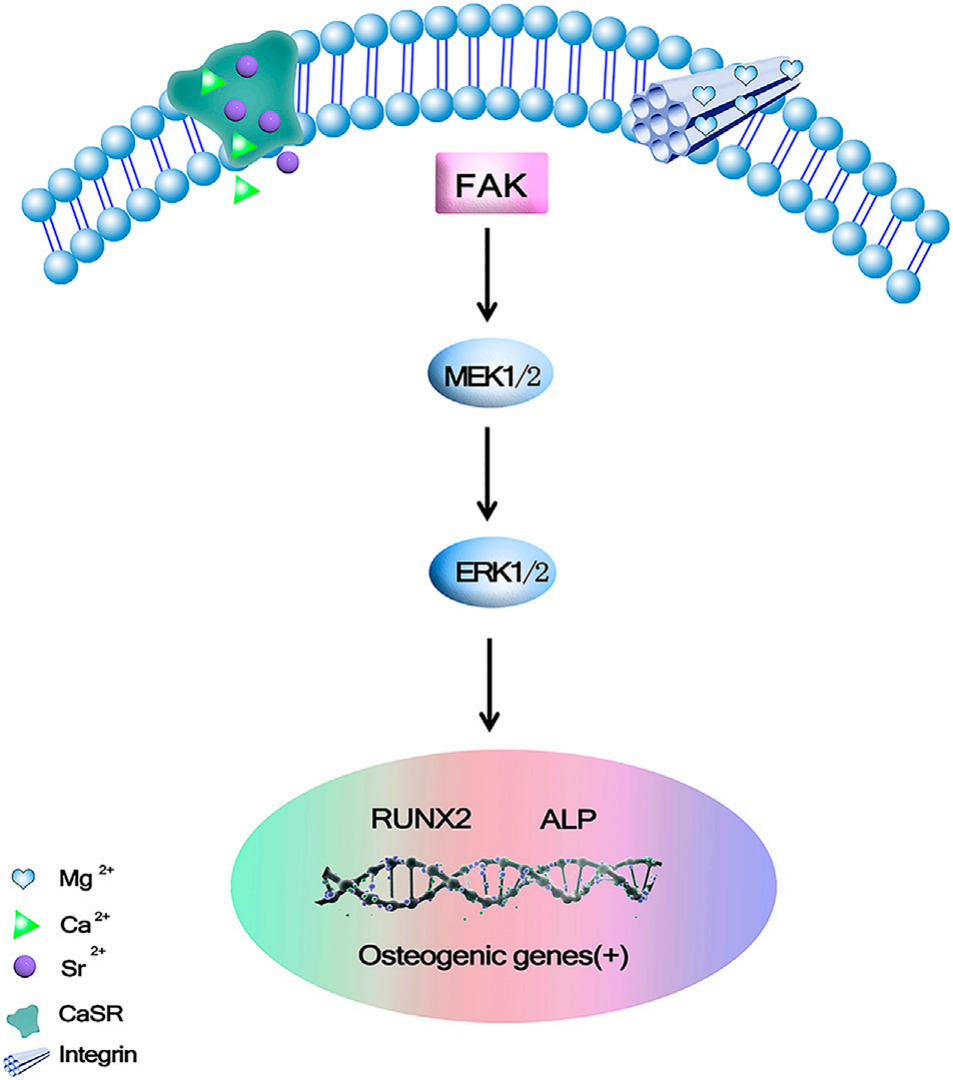
Mg^2+^, Ca^2+^ and Sr^2+^could promote osteogenic gene expression *via* binding to calcium-sensing receptor (CaSR) and activating ERK signaling pathway through Ras/Raf/MEK/ERK pathway (Li et al., 2016).

In osteoblasts, a critical member of mitogen-activated protein kinase cascades (MAPK) plays an important role in cell proliferation and differentiation. ERK1/2 is a positive regulator for osteoblast differentiation and bone formation. Studies have shown that C3G may act through ERK1/2 signaling pathways in various cells (Hu et al., 2021; Ibarra et al., 2021). Li et al. (2016) developed ternary Mg-1Ca-xwt% Sr (x = 0.2, 0.5, 1.0, 2.0) Alloys as degradable implant materials in orthopedic field. Its results showed that Mg-1Ca alloy with different amounts of Sr added during the melting process could refine the alloy grains. The extracts of the obtained cast alloys could stimulate cell viability and osteogenic differentiation, and the degree of stimulation varied according to the Sr content. The addition of 2.0 wt% Sr in Mg-1Ca alloy could induce higher proteins adsorption, and improve cell proliferation and ALP activity as a marker of osteogenic differentiation, collagen secretion, etc. ECM mineralization and osteogenesis related genes are expressed through the ERK1/2 pathway. The MAPK family is involved in regulating many cellular physiological functions, such as proliferation, differentiation, inflammation, and apoptosis, and MAPKs are indispensable factors in the process of osteoblast initiation, especially the ERK/MAPK pathway (Yang et al., 2018), in which ERK1/2 mediated the phosphorylation and transcriptional activity of Runx2 in bone (Wang et al., 2010; Park et al., 2021). Without increasing the total ERK level, it was found that Mg-1Ca-2Sr alloys induced ERK1/2 activation as well as upregulation of Runx2 expression in osteoblasts significantly, however there was no obvious difference in the amount of total and phosphorylated JNK or P38 between the treatment and control groups, and its further study found Ras/Raf/MEK of MAPK signaling pathway, ERK plays an essential role in the differentiation and maturation of osteoblasts cultured on Mg-1Ca-2Sr alloys. Figure 3 shows these alloys release Mg^2+^, Ca^2+^, and Sr^2+^. Mg^2+^ can bind to the subunits of integrins and activate the MEK/ERK pathway. Ca^2+^and Sr^2+^bind to Ca^2+^receptors (CaSR) Binding. Once the CaSR is activated by the increased divalent cation, the intracellular signaling pathways start to activate different G proteins. This leads to the activation of tyrosine kinases, phospholipase C and adenylate cyclases, which activates the ERK signaling pathway through the RAS/Raf/MEK/ERK pathway (Li et al., 2016). The Mg^2+^ integrin subunits bind, upregulate the expression of integrins in osteoblasts, and in turn activate focal adhesion kinase (FAK). FAK in integrin signal integration can directly activate ERK signaling and promote osteogenic gene expression. Berglund et al. (2017). Effects of novel Mg-Ca-Sr alloys on cellular mechanisms during degradation ion concentrations of alloy degradation products were found to be specific to hMSCs had a dual effect, increasing cell proliferation and possibly inducing osteogenesis, the effect on proliferation was attributed to the presence of Sr, whereas the addition of Mg appeared to induce osteogenesis. HMSCs differentiation was examined by changes in ALP and Runx2 genes expression, and further concluded that alloy degradation products affected the osteogenic process. ALP gene expression in osteogenic induced cultures showed that the alloy extracts had different effects on osteogenesis.

### The Underlying Mechanism of Mg Ions Promoting Osteogenesis May be Achieved by Activating the MAPK/ERK Signaling Pathway or Promoting Osteoblast Proliferation and Differentiation *via* Wnt/β - Catenin

**FIGURE 4:**
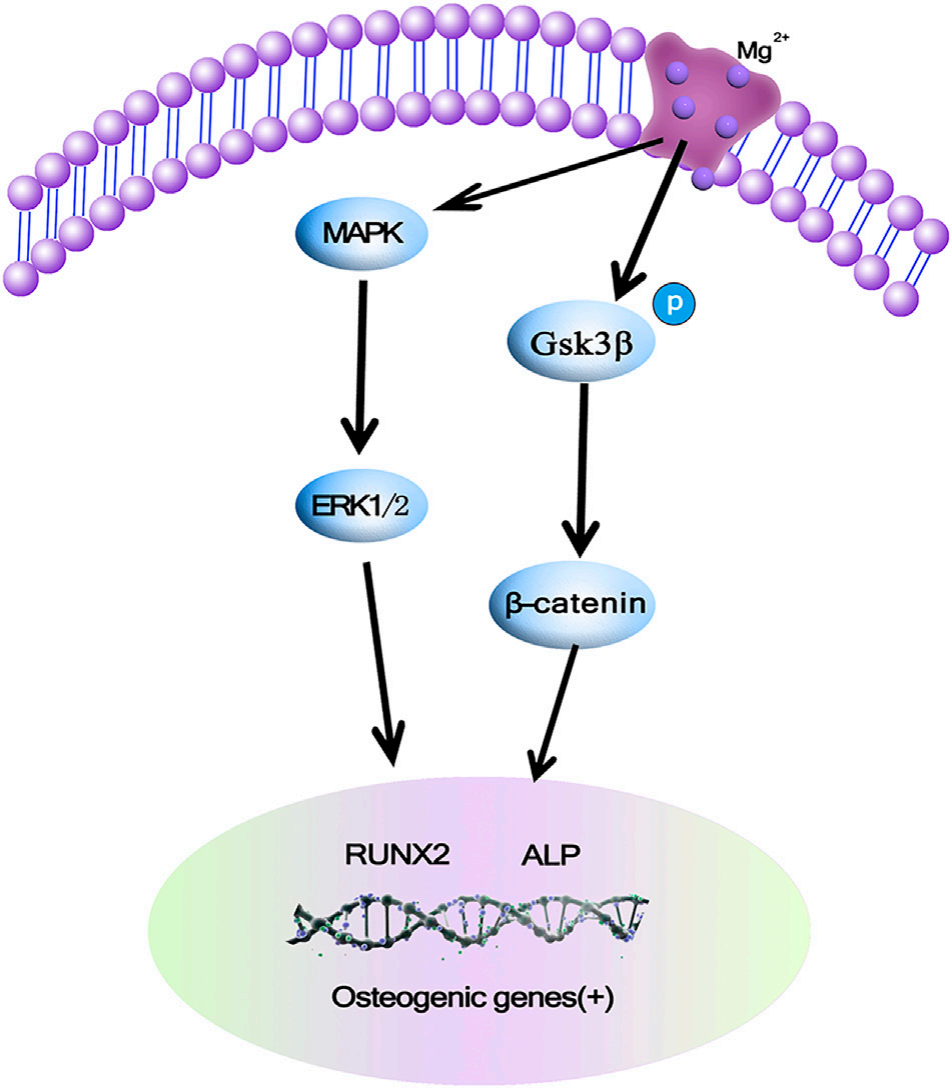
Mg ions promoting osteogenesis may be achieved by activating the MAPK/ERK signaling pathway or promoting osteoblast proliferation and differentiation *via* Wnt/β-Catenin (Iezaki et al., 2016).

MAPK/ERK is an important signaling pathway that regulates the processes of bone development, bone remodeling, and bone metabolism (Ishii et al., 2019). The main pathways of MAPK/ERK signaling include β-Catenin and GSK-3β. β-Catenin is a positive regulatory protein, which is able to promote proliferation and differentiation of osteoblasts. GSK-3β acts as a signaling protein and transcription factor to monitor cell growth (Liu et al., 2011). β-Catenin is an important positive regulator of osteoblasts and promotes osteoblast differentiation and bone formation, and several studies have also suggested different observations on the effects of Mg^2+^ on the β-Catenin pathway, which may be due to different cells. The current study gives an explanation that Mg exerts anabolic effects on bone, and in this study, Mg^2+^increased ERK phosphorylation and then enhanced the level of c-fos may help promote the proliferation of MC3T3 cells, in addition Mg^2+^ induced GSK3β phosphorylation, so the level of β-Catenin was increased due to the Mg^2+^-induced phosphorylation of GSK3β and Ca^2+^impedes GSK3β binding to β-Catenin in order to increase the level of β-Catenin and stimulate β-Catenin to form new bone. *In vitro* studies have shown that at least two pathways are involved in Mg^2+^ stimulation of bone formation, as shown in Figure 4 (Iezaki et al., 2016).

### Mg Ions is Involved in the PI3K/Akt Signaling Pathway Through the Ion Channel Functional Protein Kinase TRPM7

Two cation channels of the transient receptor potential hormone (TRPM) family have been characterized as Mg^2+^ transporters: TRPM6 and TRPM7. TRPM7 is both an ion channel and a phosphokinase active transmembrane protein, it has been implicated in cell proliferation and cell motility in various normal tissues (Yang et al., 2013; Na et al., 2018). As a divalent cation channel, TRPM7 participates in the process of cellular uptake of Mg^2+^ in the external environment, TRPM7 is also involved in Mg^2+^ absorption, and the absence of TRPM7 kinase activity can lead to the same phenotype as hypomagnesemia, which indicates that Mg^2+^ can activate TRPM7 kinase activity to mediate downstream signaling pathways and regulate cell biological functions and activities (Zhao et al., 2019). In many tissues, TRPM7 mediates the PI3K signaling pathway to regulate the proliferative activity of cells and increase the extracellular Mg^2+^ content, which relieves the growth inhibition caused by TRPM7 gene deletion. Zhang et al. (2017) human studies have confirmed that Mg^2+^promote bone repair by recruiting osteoblasts and promoting osteogenesis in osteoblasts. Inhibition of TRPM7 gene expression by siRNA gene interference found that Mg^2+^ induced ossification, mineralization migration, and chemotaxis were significantly inhibited after TRPM7 knockdown, demonstrating that the TRPM7 gene mediates the osteogenic induction by Mg^2+^ in human osteoblasts. Its data suggest that Mg ions induce osteogenesis byTRPM7/PI3K signaling upregulates the expression of Runx2 and ALP, which are markers of osteogenic differentiation, and can significantly improve the osteogenic activity of human osteoblasts. Furthermore, TRPM7/PI3K signaling increases MMP2, MMP9, and vascular endothelial growth factor (VEGF). The expression level of PI3K, a key signal transduction molecule downstream of TRPM7, induces osteoblasts from a low to a high Mg^2+^ environment by inducing cell migration. It can activate Akt protein and regulate cell proliferation, differentiation, apoptosis, and glucose transport in many tissues (Guntur and Rosen, 2011). The PI3K/Akt signaling pathway plays a crucial role in osteogenesis, and Akt phosphorylation upregulates the Runx2 gene, which induces genes associated with bone repair and remodeling, such as osteocalcin, type I collagen, osteopontin, and ALP. (Choi et al., 2014; Wang et al. (2017b)) incubation of rat calvarial osteoblasts with three different concentrations of Mg ions (6, 10, and 18 mM) revealed that appropriate concentrations of Mg^2+^ (6–10 mM) promoted osteoblast viability and differentiation, whereas excessively high Mg concentrations (18 mM has an inhibitory effect on osteoblast viability and differentiation. Blocking the PI3K/Akt signaling pathway using wortmannin, a specific inhibitor of the PI3K/Akt signaling pathway, wortmannin significantly attenuated the enhancement of cell adhesion, cell viability, ALP activity, ECM mineralization, and osteogenesis related gene expression induced by 10 mM Mg^2+^. Our study showed that 6 and 10 mM Mg^2+^ exerts stimulatory effects on cell adhesion, cell viability, ALP activity, ECM mineralization, and expression of osteogenesis related genes, and this process has been attributed, at least in part, to the activated PI3K/Akt signaling pathway.

### Mg Ions Promote Osteogenic Differentiation *via* the Notch Signaling Pathway

**FIGURE 5:**
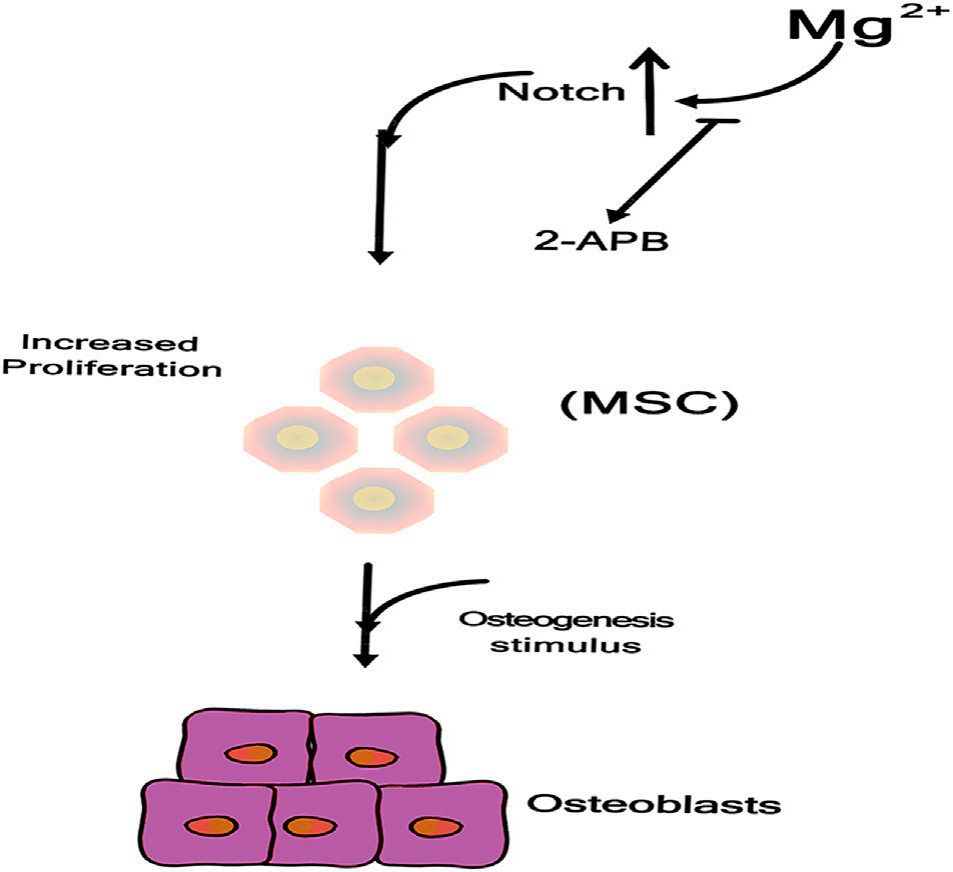
Mg ions enter into MSC through TRPM7 channel, increasing Notch Intracellular Domain (NICD) nuclear translocation (Díaz-Tocados. et al., 2017).

As shown in Figure 5, these effects were not observed after inhibition of the Mg^2+^ channel TRPM7 by 2-APB confirming the crucial role of intracellular Mg^2+^ during MSCs osteogenesis, and Mg supplementation enhances MSCs proliferation. Mechanistically, Mg ions enter MSCs through TRPM7 channels and increase Notch intracellular domain (NICD) nuclear translocation. Proliferation of MSCs contributes to subsequent osteogenesis, and inhibition of 2-APB channels decreases the osteogenic potential of Mg (Díaz-Tocados. et al., 2017; Niapour et al., 2019).

### Activation of CREB1 and SP7 by Mg Mediated CGRP

**FIGURE 6:**
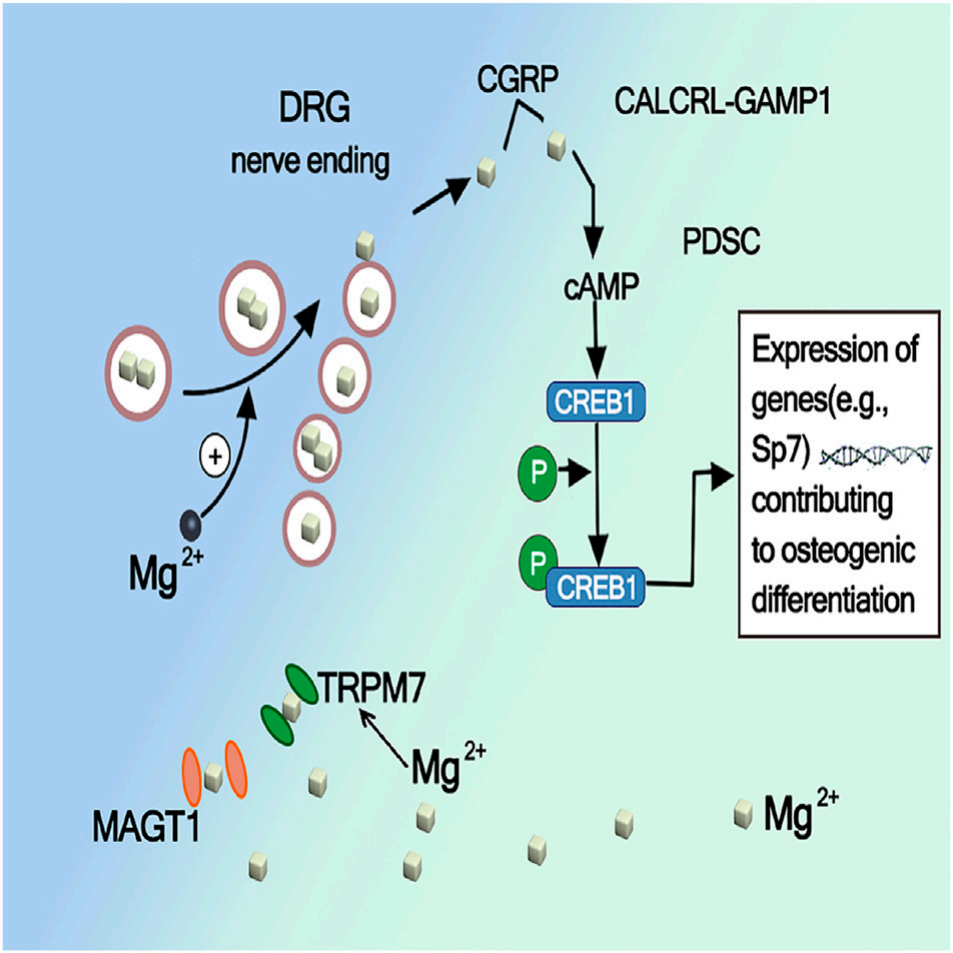
Activation of CREB1 and SP7 by Mg mediated CGRP (Zhang et al., 2016)_._

Calcitonin peptide (CGRP) primarily located in small sensory fibers, most usually found in nerves that are closely associated with blood vessels. The periosteum contains sensory nerves and releases neuropeptides, such as CGRP, which are known as involving in bone healing (). It has been demonstrated that magnesium ion release implantation enhances the synthesis of CGRP in the dorsal root ganglion (DRG) and its release at the end of the sensory nerve periosteum, and the CGRP mediated pathway has been identified as a major mechanism of potential magnesium to promote bone formation in fracture healing. Some study also demonstrates that CGRP processes an equal effect on migration and tube formation compared with VEGF, accompanied by increased VEGF expression, and activation of focal adhesion kinase (FAK), thereby acting as a strong proangiogenic growth factor during bone healing (Sun et al., 2020; Ye Li et al., 2021). Zhang et al. (2022) demonstrated through *in vitro* and *in vivo* studies that the promotion of bone formation by Mg is largely mediated by CGRP released from the sensory neuron terminals of the periosteum of the long bone axis, CGRP mediated periosteal nerves, and periosteal stem cells. The crosstalk pathway between them was identified as the main mechanism underlying Mg induced bone formation. Mg^2+^enters neurons through the ion channels MAGT1 and TRPM7 and promotes CGRP vesicle clustering and secretion in spinal root ganglion phosphorylates cyclic adenosine monophosphate (cAMP) by coupling with the CGRP receptor on periosteal stem cells, which in turn activates CREB1 and SP7 (transcription factors essential for osteogenesis) to promote osteogenesis. As shown in Figure 6, CGRP or Mg implants only upregulate SP7 and not Runx2, another downstream target of CREB1 important for bone formation, which suggests that specific activation of CREB1 and SP7 leads to the differentiation of osteoblasts *in vitro* and *in vivo*.

## Conclusion

In conclusion, The synthesis of Mg matrix composites is a hot research topic nowadays, and the host immune response is very important for bone bonding after biomaterial implantation. By studying the immune regulation of bone biomaterials, it can regulate the immune response in the process of osteogenesis, create a good local immune microenvironment, and help biomaterials reduce inflammatory response and promote good bone bonding. This study provides basic evidence for the development or modification of advanced Mg-based bone biomaterials and suggests new strategies. It has broad prospects and application value in the field of bone repair. It will provide experimental basis for exploring new bone tissue engineering materials with good antibacterial activity, excellent bone conductivity and suitable biodegradability, and further improve the application effect and scope of guided bone regeneration technology and bone tissue engineering materials in the field of implantation, It lays a good foundation for the final development of international advanced domestic bone tissue engineering materials.
